# High-Porosity Sieve-Type Neural Electrodes for Motor Function Recovery and Nerve Signal Acquisition

**DOI:** 10.3390/mi15070862

**Published:** 2024-06-30

**Authors:** Wonsuk Choi, HyungDal Park, Seonghwan Oh, Seonho Seok, Dae Sung Yoon, Jinseok Kim

**Affiliations:** 1 Center for Bionics, Korea Institute of Science and Technology, Seoul 02792, Republic of Korea; wonsuk@kist.re.kr (W.C.); hyungdal@kist.re.kr (H.P.); emart11@kist.re.kr (S.O.); 2 School of Biomedical Engineering, Korea University, Seoul 02841, Republic of Korea; 3 Center for Nanoscience and Nanotechnology (C2N), University-Paris-Saclay, 91400 Orsay, France; seonho.seok@u-psud.fr

**Keywords:** sieve-type neural electrode, nerve regeneration, motor function recovery, electrode porosity, neural signal acquisition, Sciatic Function Index (SFI)

## Abstract

In this study, the effects of electrode porosity on nerve regeneration and functional recovery after sciatic nerve transection in rats was investigated. A sieve-type neural electrode with 70% porosity was designed and compared with an electrode with 30% porosity. Electrodes were fabricated from photosensitive polyimide and implanted into the transected sciatic nerves. Motor function recovery was evaluated using the Sciatic Function Index. The number of active channels and their signal quality were recorded and analyzed to assess the sensory neural signal acquisition. Electrical impedance spectroscopy was used to evaluate the electrode performance. The group implanted with the 70% porosity electrode demonstrated significantly enhanced nerve regeneration and motor function recovery, approaching control group levels by the fifth week. In contrast, the group with the 30% porosity electrode exhibited limited improvement. Immunohistochemical analysis confirmed extensive nerve fiber growth within the 70% porous structure. Moreover, the 70% porosity electrode consistently acquired neural signals from more channels compared to the 30% porosity electrode, demonstrating its superior performance in sensory signal detection. These findings emphasize the importance of optimizing electrode porosity in the development of advanced neural interfaces, with the potential to enhance clinical outcomes in peripheral nerve repair and neuroprosthetic applications.

## 1. Introduction

Neural electrodes are critical components for interfacing the nervous system with electronic devices, facilitating the recording and stimulation of neural activity [1,2,3], and are essential in applications such as neuroprosthetics, neuromodulation, and neural circuit studies [4,5]. Peripheral neural interfaces (PNIs) are particularly important for accessing peripheral nerves directly and crucial for restoring motor and sensory functions in individuals with limb loss or nerve injuries [6,7,8]. 

Various types of neural electrodes have been developed, each designed to satisfy specific neural-interfacing requirements. Cuff electrodes, which wrap around the nerve, provide a less invasive means to measure electrical potentials, but may lack the spatial resolution required for precise neural recordings [9,10,11,12,13,14]. Longitudinal intrafascicular electrodes (LIFE) penetrate the nerve to record or stimulate individual nerve fascicles, offering high spatial resolution at the cost of significant nerve damage [15,16,17]. Transverse intrafascicular multichannel electrodes also penetrate the nerve but are designed to record from multiple sites, providing detailed neural activity mapping with invasiveness similar to that of LIFE electrodes [18,19,20]. Flat interface nerve electrodes flatten the nerve to increase the electrode contact area and improve recording stability and spatial resolution, although they can alter nerve morphology and function owing to their compressive design [21,22,23]. Flexible neural interfaces are designed to adapt to nerve movements and reduce mechanical stress and long-term damage, making them particularly useful for chronic applications where biocompatibility and stability are crucial [24,25,26]. 

**Figure 1:**
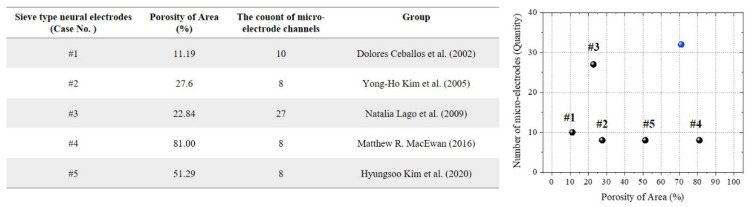
Porosity and microelectrode channel count of various sieve-type neural electrodes: This summarizes the porosity and microelectrode channel count of various sieve-type neural electrodes reported in prior studies. The table details specific designs, while the graph visualizes these data, with the blue circle representing the electrode proposed in this study [33,34,35,36,37].

Sieve-type neural electrodes, a specific type of regenerative electrode, incorporate holes that allow nerve fibers to grow, facilitating natural nerve regeneration while providing high-resolution recordings [27,28,29]. These electrodes offer several advantages, including high spatial resolution, which allows for the precise recording and stimulation of individual nerve fibers, and regenerative capability owing to their porous structure, which integrates more seamlessly with biological tissue [30,31,32]. However, the implantation process and electrode structure can cause significant initial nerve damage, potentially affecting overall functionality and recovery of the nerve. Existing sieve electrodes have varied characteristics and limitations (Figure 1). For example, Sieve Electrode #1 had a porosity of 11.19% with 10 microelectrode channels, offering limited spatial resolution but stable long-term performance [33]. However, their low porosity restricts nerve regeneration. Sieve Electrode #2, with a porosity of 27.6% and eight microelectrode channels, provides moderate spatial resolution and regenerative potential, but the limited number of channels may reduce the granularity of nerve signal recordings [34]. Sieve Electrode #3, exhibiting a porosity of 22.84% and 27 microelectrode channels, achieves higher spatial resolution and better nerve interfacing, but its increased complexity and potential for greater nerve damage during implantation are drawbacks [35]. Sieve Electrode #4, with the highest porosity of 81.00% and eight microelectrode channels, significantly enhanced nerve regeneration but potentially compromised structural integrity and electrode stability [36]. Sieve Electrode #5, featuring a porosity of 51.29% and eight microelectrode channels, balances nerve regeneration and structural support but may still induce some degree of nerve compression and damage [37].

**Figure 2:**
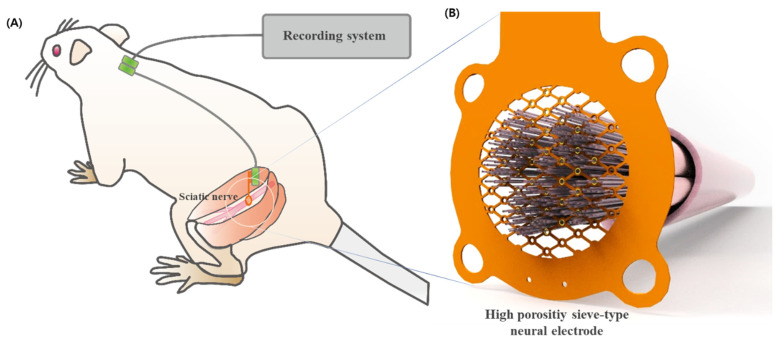
Overview of the high-porosity, sieve-type neural interface: (**A**) Schematic illustration of a rat with a high-porosity sieve-type neural electrode implanted in the sciatic nerve, connected to the recording system. (**B**) Magnified view of the neural electrode, highlighting the arrangement of the regenerative nerve fibers within the interface.

In this study, a sieve-type neural electrode with 70% porosity designed to optimize nerve regeneration and functional recovery (Figure 2) is proposed. The electrode included 32 working electrodes and three reference electrodes, which were strategically distributed to enhance the recording accuracy and minimize impedance. The high-porosity design supports more effective nerve regeneration, while the increased number of electrodes improves the spatial resolution and signal acquisition capabilities. This design aims to address the limitations of sieve electrodes by balancing regenerative support with electrical performance, thereby enhancing nerve regeneration and improving neural interfacing capabilities in peripheral nerve applications. 

## 2. Materials and Methods

### 2.1. Neural Electrode Design

**Figure 3:**
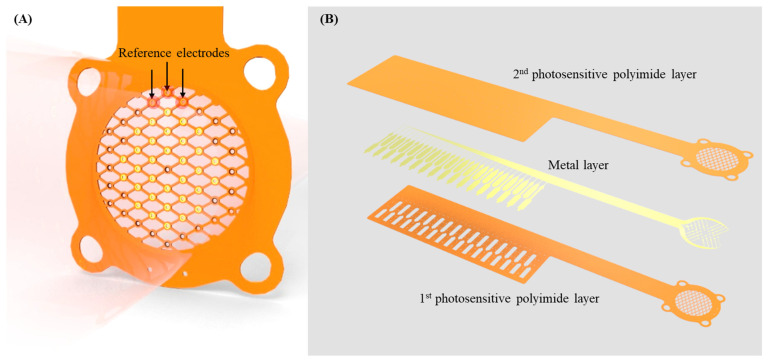
Design and layer composition of the high-porosity sieve-type neural electrode: (**A**) Sieve design over the 70% porosity area, which supports nerve regeneration. The internal arrangement includes a ring-shaped configuration with 32 working electrodes (gold) and three reference elec-trodes (black arrows). (**B**) Electrode’s layered structure, featuring photosensitive polyimide (PSPI), and metal layers of titanium/gold/titanium.

The neural electrode was designed considering the anatomical dimensions and regeneration capabilities of rat sciatic nerves, which are approximately 1.5 mm in diameter in adult rats weighing over 300 g. The design featured four suture holes at the edges for secure attachment to the transected nerve stumps. The core design was a sieve-like structure with two distinct zones. The first zone, located closer to the center of the nerve, contains circular holes each 40 µm in diameter arranged in a ring pattern. The second zone had rhombus-shaped holes aligned diagonally to promote directional nerve growth. This layout maximizes the total hole area relative to the electrode’s diameter, achieving a 70% porosity. The neural electrode included 32 working electrodes, each formed within metal rings with an outer diameter of 60 µm and an inner diameter of 40 µm, symmetrically distributed around the center. Additionally, one reference electrode identical in shape to the working electrodes was positioned along the perimeter and connected to a single circuit (Figure 3A). Connectivity between these components is facilitated by 30 µm wide bridges, which can accommodate up to two feed lines. The electrodes are connected to the electrode’s contact pad through 10 µm wide feed lines, ensuring robust electrical communication while minimizing impedance and signal loss.

### 2.2. Neural Electrode Fabrication

A high-porosity sieve-type neural electrode was fabricated from photosensitive polyimide (PSPI; HD4100, HD MicroSystems, Northbrook, IL, USA), a material commonly used in flexible neural devices (Figure 3B). To begin the fabrication process, a tri-layer of chromium, copper, and chromium (500 Å/5000 Å/500 Å, respectively) was deposited onto a 4-inch silicon wafer using an e-beam evaporator. This served as a sacrificial layer to facilitate detachment of the neural electrode from the substrate. The first layer of PSPI was spin-coated at 3000 rpm to achieve a thickness of 7 µm. The PSPI layer was then exposed using a mask aligner (MA 6, Karl Suss, Garching, Germany), except in the areas designated as the working and contact pads. This was followed by full curing at 350 °C in a vacuum oven for 2 h in an oxygen-free environment. To enhance the adhesive properties between PSPI and the subsequent metal layers [38], oxygen plasma ashing was performed at 100 watts for 30 s. Subsequently, metal deposition was conducted in the order of titanium, gold, and titanium (200 Å/3000 Å/200 Å) from the bottom to the top using an e-beam evaporator. A photoresist (GXR-601 46cp, AZ Electronics Materials, Darmstadt, Germany) was then patterned to mask the deposited metal layer before dry etching. The working electrodes and 33 metal feedlines, comprising 32 working electrodes and one reference electrode, were formed by dry etching using an inductively coupled plasma reactive-ion etcher (ICP-RIE, Oxford Instruments, Oxford, UK). The second PSPI layer was then applied to a thickness of 7 µm to passivate and encapsulate the metal structures, providing support for the entire neural electrode. This layer was processed under the same conditions as the first layer. Finally, the entire sieve-type neural electrode was released from the wafer by dissolving the sacrificial layer in a chromium etchant (CR-7, Cyantek Corporation, Fremont, CA, USA) at 27 °C. The electrodes were then rinsed with deionized water and dried. The fabricated neural electrodes were examined using an optical microscope (Meiij EMZ, MEIJI TECHNO CO. LTD, Saitama, Japan) to observe their enlarged form. For a more precise examination of the working electrode’s shape, a scanning electron microscope (SEM) (Inspect F50, ELECMI, Estepona, Spain) was used under an accelerating voltage of 15 kV to verify the electrode structure. This fabrication process ensured the structural integrity and functional efficacy of the high-porosity sieve-type neural electrode, making it suitable for neural interface applications. 

### 2.3. In-Vivo Experimentation—Electrode Implantation Surgery

Nine male Sprague–Dawley rats, each weighing approximately 300 g, were used in this study. The animals were divided into three groups based on the experimental procedure applied to the sciatic nerve. The first group (TG) underwent transection, followed by sciatic nerve reattachment. The second group (70S) underwent sciatic nerve transection, followed by the implantation of a sieve-type neural electrode with 70% porosity. The third group (30S) underwent a similar transection, followed by the implantation of a sieve-type neural electrode with 30% porosity. All surgical procedures were conducted under deep anesthesia, induced by an intramuscular injection of a 3:1 mixture of zolethyl and rumpun, at a dosage of 0.08 mL per 100 g of body weight. To expose the sciatic nerve, an incision was made in the gluteal muscle of the biceps femoris in the right thigh. After exposure, the sciatic nerve was transected. Subsequently, the respective procedures for nerve reattachment and electrode implantation were performed according to group assignments. All experimental protocols were approved by and conducted in accordance with the ethical guidelines of the Institutional Animal Care and Use Committee (IACUC) of the Korea Institute of Science and Technology (KIST), under the approval number: KIST-IACUC-2022-015-2. This ensured ethical handling and welfare of the animals throughout the study.

### 2.4. In-Vitro Impedance Testing Using Impedance Analyzer

Before surgical implantation of the high-porosity sieve-type neural electrode, its electrical characteristics were assessed in vitro. Impedance measurements were conducted in a phosphate-buffered saline (PBS) solution using a three-electrode setup. The working electrodes of the sieve were connected to Ag/AgCl reference electrodes, and a Pt wire served as the counter electrode. The impedance and phases of all 32 working electrodes were analyzed across a frequency range of 10 Hz to 100 kHz with a 20 mV RMS amplitude, employing a VersaSTAT3 potentiostat (AMETEK, Berwyn, PA, USA). This method ensured an accurate evaluation of the electrical properties of the electrode, which is essential for effective neural signal recording and transmission.

### 2.5. Sciatic Function Index (SFI) for Motor Function Recovery

Motor function recovery analysis was conducted using a walking track covered with paper, measuring approximately 20 cm wide and 100 cm long. The analysis was performed preoperatively and weekly from 1 to 8 weeks postoperatively. Blue ink was applied to the hind paws of the experimental subjects in each of the three groups to track paw placement and gait during walking. Footprints were scanned, digitized, and analyzed to calculate Sciatic Function Index (SFI). The SFI is designed to noninvasively monitor nerve regeneration following nerve crush or transection. Sciatic nerve damage was quantitatively assessed by gait trajectory analysis. For gait analysis, the following measurements were obtained from the rat footprints: print length (PL), which is the distance from the heel to the third toe; toe spread (TS), which is the distance from the first to the fifth toe; and intermediate toe spread (ITS), which is the distance from the second to the fourth toe. These measurements were obtained from both experimental (E) and normal (N) sides. SFI was calculated using the following formula:

SFI = −38.3(EPL-NPL)/NPL + 109.5(ETS-NTS)/NTS + 13.3(EITS-NITS)/NITS − 8.8

In the control group with healthy nerves, the SFI was expected to be approximately zero, indicating normal function. In the experimental groups where the sciatic nerve was transected and reattached with the sieve-type neural electrode, the SFI was anticipated to be approximately −100, indicating complete dysfunction. This comparison highlighted the extent of motor function recovery under different experimental conditions.

### 2.6. Neural Signal Recording

Sensory signal recordings were performed every two weeks post-surgery to assess nerve recovery in all subjects implanted with 70% and 30% porosity sieve-type neural electrodes. The rats were anesthetized using isoflurane inhalation anesthesia, and tactile stimulation was applied to the entire sole using a 10 mm wide brush at a consistent speed and interval. This procedure aims to elicit afferent sensory nerve signals as nerve recovery progresses after electrode implantation. Neural signals were recorded using the back-end pads of the electrodes connected to a flexible printed circuit (FPC) connector (Molex, Lisle, IL, USA). This FPC connector was interfaced with an omnetic connector through 34 electrodes, consisting of 32 working electrodes, one reference electrode connected to three ring-shaped electrodes, and one body ground electrode (Au-coated fabric) positioned around the surrounding muscles for in vivo electromyography shielding, each wire was 15 cm in length. The signal recorded by each working electrode, including channel 32, was differentially compared with the reference electrode. The reference electrode consisted of three ring-shaped electrodes linked together to form a single reference point. Positioned near the epineurium, the outermost layer of the nerve, this strategic placement allowed for differential comparison between the signal recorded by the working electrodes and the reference electrode near the nerve fibers within the nerve. As a result, neural signals from the nerve fibers could be accurately acquired through this differential method. The wires were routed subcutaneously to the back of the neck, where the omnetic connector was externalized through the skin. The externalized connector was connected to an Intan RHS 32-Channel Stim/Recording Headstages (Intan Technologies, Los Angeles, CA, USA) and a serial peripheral interface (SPI) cable from a stimulation/recording controller to enable signal capture and stimulation. Neural signals were recorded using an Intan stimulation/recording controller equipped with proprietary software. The recording settings were carefully optimized for signal clarity and integrity. The amplifier was sampled at a rate of 30 kS/s, with a cutoff frequency of 300 Hz. The bandwidth filters were adjusted to a low of 30 Hz and high of 5 kHz, with a high-pass filter at 250 Hz and a notch filter at 60 Hz to minimize electrical noise. The recordings specifically targeted the sensory nerve signals elicited by applying tactile stimulation to the entire sole of the hind limbs using a soft standard brush. All 32 channels of the recording device were actively monitored to identify and analyze the responses from the sensory nerves, ensuring comprehensive coverage of sensory responses and accurate assessment of the functionality and sensitivity of the implanted neural electrodes.

### 2.7. Neural Signal Analysis

Neural signal data recorded from the 32-channel electrodes in both experimental groups (70% and 30% porosity sieve-type neural electrodes) were processed and analyzed using a custom MATLAB R2015a program. To reduce noise, a Butterworth filter was applied during post-processing under the following conditions: a high-pass filter at 300 Hz, a low-pass filter at 3 kHz, and a sampling rate of 30 kS/s. Feature extraction of neural spikes was performed using a custom MATLAB program, followed by clustering of the extracted data using the k-means method. A fast Fourier transform (FFT) was employed to confirm noise filtering in the raw data from the Intan recordings. Additionally, the number of channels that successfully acquired neural signals was recorded every two weeks up to the 8th week for both experimental groups. This analysis compared the number of signal-acquired channels between the two porosity groups, highlighting the impact of electrode porosity on neural signal acquisition.

### 2.8. Immunohistochemistry for Nerve Tissue Staining

Tissue analysis was performed on the sciatic nerves of the experimental group to confirm nerve regeneration in subjects implanted with the 70% porous sieve-type neural electrode. Eight weeks post-implantation, the sciatic nerve tissue was harvested to assess the regeneration environment within the porous areas of the electrode using immunohistochemistry. First, perfusion was performed with 4% paraformaldehyde (PFA) in PBS to remove blood from the body. Subsequently, the sciatic nerve tissue, including the implanted neural electrode, was excised. Tissue samples were obtained 5 mm proximally and distally to the implant site. The extracted nerves were frozen in optimal cutting temperature (OCT) compound (FSC22; Leica Biosystems Richmond, Inc., Dublin, Ireland). To visualize neurofilaments, the sections were incubated overnight with the primary mouse anti-neurofilament antibody (ab7794, Abcam Inc., Cambridge, UK). For cell nuclear staining, the sections were washed three times in 1% BSA and then incubated with 4′,6-diamidino-2-phenylindole (DAPI) (Thermo Fisher Scientific Inc., Waltham, MA, USA) for 10 min at room temperature in the dark. Images were captured using a confocal microscope (Leica Microsystems, Wetzlar, Germany) to visualize the stained tissues, allowing assessment of nerve regeneration within the porous regions of the sieve-type neural electrode.

## 3. Results

### 3.1. Fabrication and Implantation of the Neural Electrode

**Figure 4:**
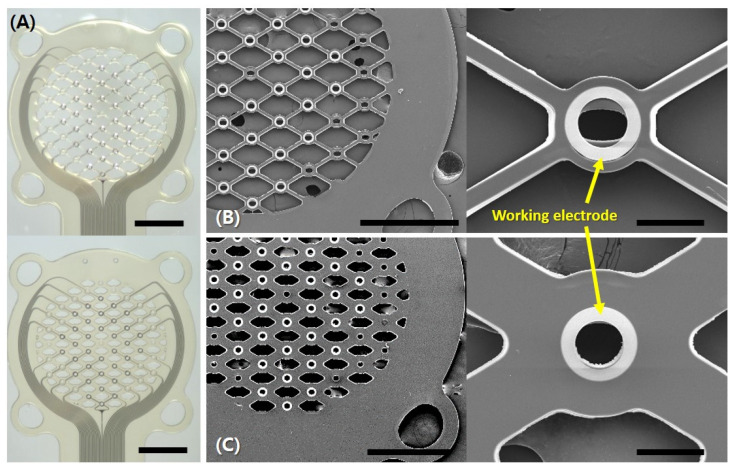
Visualization of sieve-type neural electrodes. (**A**) Optical optical images displaying sieve-type neural electrodes with 70% porosity (**up**) and 30% porosity (**down**). The scale bar is 600 µm. (**B**) Scanning electron microscope (SEM) images of the 70% porosity sieve-type neural electrode, showing the regenerative ring-shaped arrangement of the 32 working and reference electrodes. The left image scale bar is 500 µm. The right image is a magnified view of a working electrode with a scale bar of 50 µm. (**C**) SEM images detailing the structure of the 30% porosity sieve-type neural electrode. The left image scale bar is 500 µm. The right image is a magnified view of a working electrode with a scale bar of 50 µm.

**Figure 5:**
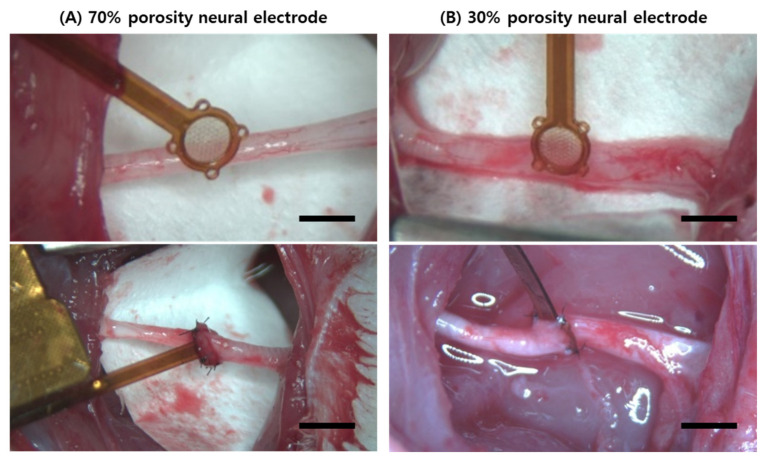
Implantation surgery of the rat sciatic nerve. (**A**) The top image shows the 70% porosity sieve-type neural electrode before implantation. The bottom image displays the electrode after sci-atic nerve transection and insertion, secured with four suture points. The scale bars are 2 mm (**top**) and 2.5 mm (**bottom**). (**B**) The top image illustrates the 30% porosity sieve-type neural electrode before implantation. The bottom image shows the electrode inserted into the transected sciatic nerve. The scale bars are 1.5 mm (**top**) and 2 mm (**bottom**).

Optical and scanning electron microscopies (SEM) were used to verify the manufacturing accuracy of the electrodes. In Figure 4A, the upper optical image shows a 70% porosity and the lower optical image shows a 30% porosity sieve-type neural electrode, highlighting the differences in porosity areas. SEM images of the 70% porosity are shown in Figure 4B and the 30% porosity sieve type is shown in Figure 4C. Both types of neural electrodes feature ring-shaped working electrodes with the same area, as shown in the right images of Figure 4B,C. The 70% porosity (Figure 5A) and 30% porosity sieve-type neural electrodes (Figure 5B) were successfully implanted by suturing the epineurium to the four suture points after transecting the right sciatic nerve perpendicular to its length.

### 3.2. Electrical Characteristics of the Neural Electrode

**Figure 6:**
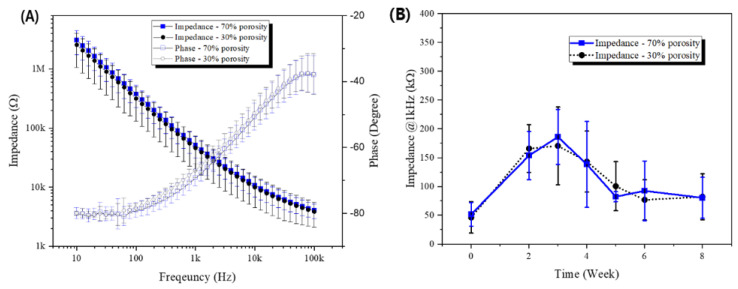
Electrical characteristics of sieve-type neural electrodes. (**A**) Graph showing the impedance and phase characteristics of the 70% porosity sieve-type neural electrode (blue square boxes and solid lines) and the 30% porosity sieve-type neural electrode (black circles and solid lines). (**B**) Graph showing the impedance recorded at a frequency of 1 kHz over an 8-week period following surgical implantation into the sciatic nerve for the 70% porosity sieve-type neural electrode (blue square boxes and solid lines) and the 30% porosity sieve-type neural electrode (black circles and dashed lines).

The electrical characteristics were assessed using electrical impedance spectroscopy (EIS). Electrical impedance tests across all channels consistently showed low values, which are essential for effective neural signal transmission. The 70% porosity sieve-type neural electrode demonstrated an impedance at 1 kHz averaging 51.6 ± 20.3 kΩ, with a phase angle of −69.3 ± 2.5°. Conversely, the 30% porosity sieve-type neural electrode showed an average impedance of 46.1 ± 27.2 kΩ and a phase angle of −67.2 ± 2.1° (Figure 6A). 

Impedance values were tracked over time following the implantation of the neural electrodes (Figure 6B). By the third week post-implantation, the average impedance for the 70% porosity sieve-type neural electrode increased to approximately 185.9 ± 47.7 kΩ, while the 30% porosity electrode increased to approximately 170.5 ± 67.4 kΩ. From the fourth week onwards, a decrease in impedance was observed, stabilizing at approximately 80.3 ± 35.6 kΩ for the 70% porosity electrode and 81.9 ± 40.0 kΩ for the 30% porosity electrode, returning to levels similar to pre-implantation measurements. The initial increase in impedance within the first three weeks is attributed to fibrotic tissue formation around the electrodes, which is a common physiological response. However, the subsequent decrease and stabilization of impedance values from the fourth week onwards suggest that the surrounding tissue is adapting to the presence of the electrodes, returning to near initial levels.

### 3.3. Motor Function Recovery

**Figure 7:**
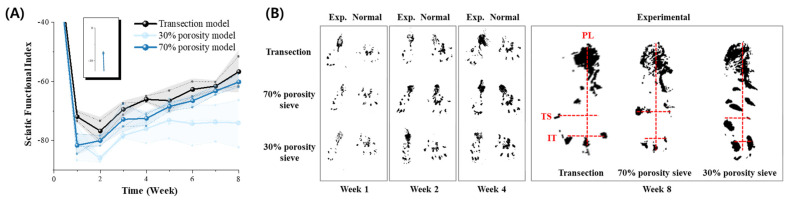
Sciatic functional index (SFI) and footprint analysis for motor function recovery. The left (**A**) graph displays the SFI over an 8-week period for the transection model (black), 30% porosity sieve-type neural electrode (light blue), and 70% porosity sieve-type neural electrode (dark blue). Error bars indicate standard deviations. The right panels (**B**) show representative footprint patterns at 1, 2, 4, and 8 weeks post-surgery. The experimental groups (transection, 70% porosity sieve, and 30% porosity sieve) are compared to normal controls. The 8-week footprints include detailed markers for paw length (PL), toe spread (TS), and intermediary toe spread (IT), illustrating the extent of functional recovery.

Motor function recovery was evaluated over 8 weeks using the Sciatic Function Index (SFI), with weekly measurements. The study included a transection group (TG) as a positive control, a 30% porosity sieve group as a negative control, and a 70% porosity sieve group. The results indicated that while the 70% porosity group did not initially match the recovery levels of the TG, they significantly improved and approached these levels starting from week five (Figure 7A). The preoperative SFI values for the three groups were as follows: TG: −7.63 ± 0.06, 30% group: −7.98 ± 0.47, 70% group: −7.74 ± 0.59. In the first and second weeks after surgery, all groups showed decreased SFI values. The SFI values for the 70% group were −81.61 and −80.01, respectively, while the 30% group’s values were −80.42 and −86.21, and the TG recorded −71.94 and −76.77, showing higher values than the electrode-implanted groups. Motor function began to recover from the third week, and the TG had SFI values of −71.94 at the third week and −56.73 at the eighth week, showing the highest recovery. The 70% porosity group showed gradual recovery with SFI values improving from −72.84 at the third week to −60.79 at the eighth week, whereas the 30% porosity group showed values improving from −78.49 at the third week to −74.22 at the eighth week, indicating slower motor function recovery (Table 1).

This finding highlights the significant role of electrode porosity in nerve regeneration and motor function recovery. Figure 7B shows the footprints of the left normal paw and right experimental paw with implanted neural electrodes at 1, 2, 4, and 8 weeks post-surgery. By the eighth week, the 70% group exhibited greater separation (TS length) between the first and fifth toes compared to the 30% group, suggesting more effective motor function recovery in the 70% porosity group. These data provide further evidence for the impact of electrode porosity on the efficacy of nerve regeneration and functional recovery post-injury.

### 3.4. Results of Neural Signal Acquisition, Sorting, and Clustering

**Figure 8:**
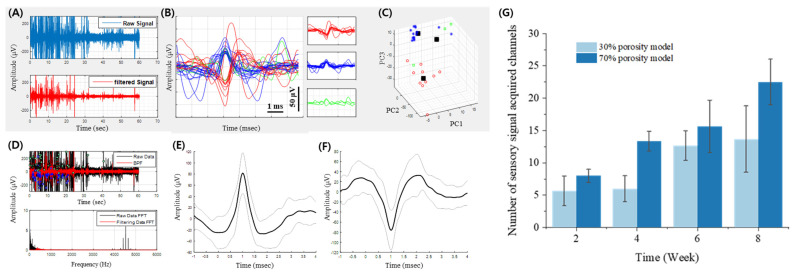
Sensory Signal Acquisition and Analysis. (**A**) Representative recordings of raw and filtered sensory signals obtained from the 70% porosity sieve-type neural electrode. These panels show time-domain signals, with the top graph displaying the raw signal in blue and the bottom graph showing the filtered signal in red. (**B**) This graph presents a multi-dimensional analysis, including the principal component analysis (PCA) plot, showing the differentiation between signal clusters in (**C**). This data is recorded from a single working electrode channel at the 2-week mark. (**D**) The graph depicts the raw data and filtered data in the time domain, with the fast Fourier transform (FFT) results on the bottom left. The graphs display averaged sensory signal waveforms and their corresponding standard deviations in (**E**) positive and (**F**) negative action potentials. This data is also from a single working electrode channel at the 2-week mark. (**G**) Bar graph comparing the number of sensory signal-acquired channels between the 30% porosity model (light blue) and the 70% porosity model (dark blue) over an 8-week period. Error bars represent standard deviations. Each bar represents the average number of active channels and their standard deviations, demonstrating that the 70% porosity model consistently achieves higher signal acquisition, indicating its superior performance in sensory signal detection.

Neural signal acquisition, sorting, and clustering were performed to evaluate the sensory signal detection capabilities of the 70% and 30% porosity sieve-type neural electrodes. Representative recordings of raw and filtered sensory signals obtained from the 70% porosity sieve-type neural electrode are shown in Figure 8A. The top panel displays the raw signal in blue and the bottom panel shows the filtered signal in red. These time-domain signals illustrated the initial neural activity captured by the electrodes. Figure 8B presents the extraction and sorting of three types of neural spikes using applied filters (scale bars: *x*-axis, 1 ms; *y*-axis, 50 μV). The multidimensional analysis, including the principal component analysis (PCA) plot shown in Figure 8C, demonstrates the differentiation between signal clusters. These data, recorded from a single working electrode channel at the 2-week mark, highlight the capability of the electrode to distinguish between various neural signals. Figure 8D shows the raw and filtered data in the time domain, with the fast Fourier transform (FFT) results shown at the bottom left. FFT analysis confirmed effective noise reduction and signal clarity achieved through filtering. The averaged sensory signal waveforms and their corresponding standard deviations for positive and negative action potentials are displayed in Figure 8E and Figure 8F, respectively (scale bars: *x*-axis, ms; *y*-axis, μV). These waveforms were also recorded from a single working electrode channel at the 2-week mark. The number of sensory signal-acquired channels was compared between the 30% porosity model and the 70% porosity models over 8 weeks, as shown in Figure 8G. The bar graph indicates that the 70% porosity model consistently achieved higher signal acquisition, with each bar representing the average number of active channels and their standard deviations. This demonstrates the superior performance of the 70% porosity model for sensory signal detection, further underscoring the importance of electrode porosity in neural interface design.

In summary, the 70% porosity sieve-type neural electrode exhibited a higher number of active channels and more effective sensory signal acquisition compared to the 30%-porosity model. This enhanced performance can be attributed to the optimized porosity, which facilitates better integration with the surrounding neural tissue and more reliable signal transmission. A detailed analysis of neural signals, including sorting and clustering, provided robust evidence of the efficacy of the electrode in capturing and distinguishing neural activity, making it a promising tool for neural interfacing applications.

### 3.5. Immunohistochemistry for Nerve Fibres

**Figure 9:**
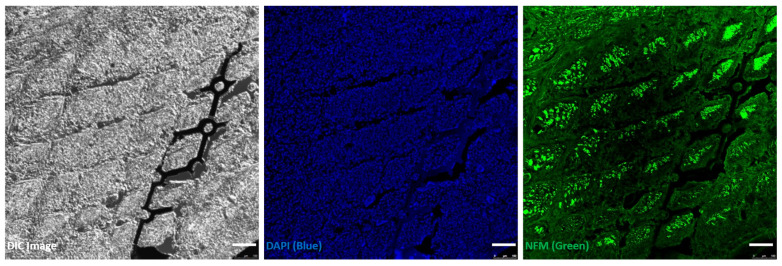
Immunohistochemistry (IHC) analysis post-implantation of the 70% porosity sieve-type neural electrode. Differential interference contrast (DIC) image (**left**), DAPI staining for cell nuclei in blue (**middle**), and neurofilament staining (NFM) in green (**right**) are shown for the sciatic nerve 8 weeks after implantation of the 70% porosity sieve-type neural electrode. The images illustrate the integration of the neural electrode with the surrounding nerve tissue, highlighting the structural integrity and cellular organization around the implant. Scale bar represents 100 µm.

Immunohistochemical (IHC) staining was performed at the 8-week mark following the implantation of the 70% porosity sieve-type neural electrode to assess nerve regeneration (Figure 9). The analysis revealed the presence of nerve fibers and cell nuclei within the spaces of the sieve pores. This observation indicated successful nerve regeneration, suggesting that the structural design of the electrode supported and perhaps enhanced neural growth and integration within the treated area. This finding is crucial as it demonstrates the potential of porous sieve electrodes to facilitate and guide nerve repair after injury.

## 4. Discussion

The mechanical stability of neural electrodes is crucial for long-term functionality and integration with neural tissue [39]. Our electrodes use a photosensitive polyimide with high tensile strength (200 MPa), elongation (45%), and modulus (3.5 GPa), maintaining integrity under stress [40,41]. Additionally, however, the tensile force from subcutaneously routed wires may cause damage over long-term implantation. Thus, developing multichannel wireless signal transmission and stimulation pulse generators is essential to prevent such damage.

After the implantation of the neural electrodes, an initial increase in impedance was observed across all channels. This was primarily attributed to the immune response triggered by the introduction of a foreign body, which led to the formation of fibrotic tissue around the electrode. Such fibrosis, as documented in the referenced literature, is typical within the first three weeks post-implantation [42,43]. The reduction in impedance observed from the fourth week may be attributed to the continuous administration of antibiotics, which likely mitigated the immune response and reduced fibrosis, and to tissue adaptation and remodeling over time, enhancing electrode–tissue integration [44]. This suggests that managing immune responses and natural tissue adaptation can improve the functional integration and long-term viability of neural electrodes.

The high porosity of the sieve-type electrode enhances nerve regeneration by allowing more nerve fibers to bridge the gap and contact the electrode surface [45]. Higher porosity levels generally support better nerve regeneration by increasing space for tissue in-growth and vascularization, facilitating critical nutrient and waste exchange for cell survival and function [46]. However, excessively high porosity can compromise spatial resolution necessary for accurate neural signal recording by reducing the density of recording sites. Our design minimizes the width of the working electrode and bridge while maximizing porosity. Through optimization, we found that a 70% porosity level provides an optimal configuration, allowing sufficient tissue integration without compromising the spatial resolution needed for effective neural signal recording. The 70% porosity in our study is based on considerations of biological integration, signal recording capability, and structural design requirements.

The acquisition and separation of sensory signals from specific channels, especially when stimulated at precise anatomical locations on the limbs of experimental subjects, indicate the need for more refined experimentation and advanced signal separation technologies [47]. The current results highlight the potential of identifying and isolating neural signals corresponding to specific stimuli. However, further development of electrode design and signal processing algorithms is required to enhance the specificity and reliability of these recordings. Future studies should focus on improving the spatial resolution of neural interfaces and refining techniques to distinguish between different types of neural activity.

The primary aim of our IHC analysis for the 70% porosity group was to verify the biocompatibility and adaptability by observing the extent to which nerve fibers regenerated and integrated into the vacant spaces of the sieve-type electrode. The IHC process was conducted to confirm the biocompatibility and safety of the proposed neural electrode design and material, rather than to compare the regeneration differences based on porosity levels. The IHC staining results for the 70% porosity group showed that by the eighth week, the nerve fibers had successfully regenerated and filled the structured spaces of the electrode. This indicates that the nerve regeneration within the electrode structure was well achieved, confirming the biocompatibility and stability of the neural electrode. We acknowledge the limitation of not having comparative data for the 30% porosity group. Future studies will aim to include comprehensive comparative analyses with various porosity levels to further substantiate these findings.

Although sieve electrodes are highly invasive, they offer a significant potential for selective nerve interfaces. Increased porosity facilitates enhanced nerve fiber contact, which is crucial for effective signal acquisition. However, the challenge remains in balancing the structural integrity of the electrode with its porosity to avoid mechanical failure during long-term implantation.

## 5. Conclusions

This study demonstrated that sieve-type neural electrodes with high porosity significantly enhanced nerve regeneration and motor function recovery following peripheral nerve injury. The 70% porosity electrode exhibited promising results. From the fifth week, the SFI suggested an improvement in recovery levels, approaching those of the control group. Additionally, IHC analysis confirmed successful nerve regeneration by the eighth week. The optimized 70% porosity design promoted natural nerve growth and effective neural signal acquisition, highlighting the importance of electrode porosity in neural interface design. The increase in the initial impedance suggests the need for strategies to manage the immune response and fibrosis. These findings underscore the critical role of electrode porosity in improving neural interfaces for clinical applications in nerve injury treatment and neuroprosthetics. However, the study also revealed limitations, such as the initial increase in impedance due to fibrotic tissue formation and the potential mechanical fragility of high-porosity electrodes. Future studies should focus on elucidating the mechanisms by which porosity affects tissue responses and on optimizing electrode designs to balance porosity with mechanical stability. Additionally, the development of advanced materials and coatings to mitigate immune responses and fibrosis could enhance long-term electrode performance and integration.

## Figures and Tables

**Table 1:** Sciatic functional index (SFI) scores post-surgery for different neural electrode models. The table presents the mean and standard deviation of SFI scores for the transection model, 30% po-rosity model, and 70% porosity model over an 8-week period post-surgery.

		After Surgery	1	2	3	4	5	6	7	8
Transection group	Mean	−7.639	−71.949	−76.772	−69.488	−66.074	−66.509	−62.734	−61.607	−56.731
	Standard deviation	0.068	2.276	4.587	2.764	1.326	4.606	3.588	1.848	7.136
30% porosity group	Mean	−7.981	−80.423	−86.212	−78.499	−76.202	−73.289	−74.483	−73.989	−74.224
	Standard deviation	0.474	5.840	0.982	1.140	4.174	6.830	6.829	6.181	7.977
70% porosity group	Mean	−7.744	−81.619	−80.007	−72.843	−72.510	−68.482	−66.560	−63.243	−60.199
	Standard deviation	0.593	3.405	1.792	4.411	2.353	1.512	2.062	1.427	2.251

## Data Availability

The original contributions presented in the study are included in the article, further inquiries can be directed to the corresponding authors.
